# Effects of colour-coded compartmentalised syringe trays on anaesthetic drug error detection under cognitive load

**DOI:** 10.1016/j.bja.2023.12.033

**Published:** 2024-02-09

**Authors:** Victoria Laxton, Frances A. Maratos, David W. Hewson, Andrew Baird, Stephanie Archer, Edward J.N. Stupple

**Affiliations:** 1 Automation, TRL, Crowthorne, UK; 2 School of Psychology, College of Health, Psychology and Social Care, University of Derby, Derby, UK; 3 Department of Anaesthesia, Academic Unit of Injury, Recovery and Inflammation Science, School of Medicine, University of Nottingham, Nottingham, UK; 4 Nottingham University Hospitals NHS Trust, Nottingham, UK; 5 Department of Psychology, University of Cambridge, Cambridge, UK; 6 Department of Public Health and Primary Care, University of Cambridge, Cambridge, UK; 7 Faculty of Medicine, Department of Surgery and Cancer, Imperial College London, London, UK

**Keywords:** cognitive load, colour coding, injectable, medication error, syringe trays, visual search

## Abstract

**Background:**

Anaesthetic drug administration is complex, and typical clinical environments can entail significant cognitive load. Colour-coded anaesthetic drug trays have shown promising results for error identification and reducing cognitive load.

**Methods:**

We used experimental psychology methods to test the potential benefits of colour-coded compartmentalised trays compared with conventional trays in a simulated visual search task. Effects of cognitive load were also explored through an accompanying working memory-based task. We hypothesised that colour-coded compartmentalised trays would improve drug-detection error, reduce search time, and reduce cognitive load. This comprised a cognitive load memory task presented alongside a visual search task to detect drug errors.

**Results:**

All 53 participants completed 36 trials, which were counterbalanced across the two tray types and 18 different vignettes. There were 16 error-present and 20 error-absent trials, with 18 trials presented for each preloaded tray type. Syringe errors were detected more often in the colour-coded trays than in the conventional trays (91% *vs* 83%, respectively; *P*=0.006). In signal detection analysis, colour-coded trays resulted in more sensitivity to the error signal (2.28 *vs* 1.50, respectively; *P*<0.001). Confidence in response accuracy correlated more strongly with task performance for the colour-coded tray condition, indicating improved metacognitive sensitivity to task performance (*r*=0.696 *vs r*=0.447).

**Conclusions:**

Colour coding and compartmentalisation enhanced visual search efficacy of drug trays. This is further evidence that introducing standardised colour-coded trays into operating theatres and procedural suites would add an additional layer of safety for anaesthetic procedures.

**Editor's key points**

• Medication errors are common in anaesthesia such that systems approaches to mitigation can have significant impact on patient safety.
• Colour-coded anaesthetic drug trays could increase error identification and reduce cognitive load.
• This simulation study in volunteer anaesthesia practitioners examined the impact of colour-coded drug compartment trays on drug-detection error and search time under cognitive load.
• Colour coding and compartmentalisation enhanced visual search efficacy of drug trays, which could add an additional layer of safety in anaesthesia procedures.

Drug administration during anaesthesia is complex and commonly occurs concurrent with fatigue, stress, and high cognitive load. Anaesthetists frequently work in noisy, cluttered, and time-pressured environments. Multitasking (e.g. conversations during drug preparation) disrupts working memory,^1^, ^2^, ^3^, ^4^ deflects attention from the primary task, and results in action slips.^5^, ^6^, ^7^

Drug errors have been reported to occur in at least one in 133 procedures,^8^^,^^9^ with syringe swaps (a syringe mistakenly administered in place of another^10^^,^^11^) accounting for 81% of mistakes.^12^ As these errors often involve drugs with powerful cardiorespiratory and neuromuscular effects,^13^^,^^14^ mitigation strategies could improve perioperative patient safety. Physical grouping of information (compartments) and use of visual cues (colour coding) can reduce cognitive load,^15^, ^16^, ^17^ and has been applied to support airway management.^18^ An eye-tracking study showed the benefits of a colour-coded tray with standardised drug locations, improving visual search efficacy and enabling rapid error identification compared with conventional trays.^19^

Situational awareness is essential for task performance.^20^ Given that perceptual errors are common, and errors of situational comprehension can follow from perceptual errors, simplifying perceptual tasks to ameliorate information processing is desirable in safety critical environments.^20^ If colour-coded compartmentalised trays enable faster visual search while preserving or enhancing error detection, this would facilitate perceptual situational awareness. Simplifying visual search might also enable the wider theatre team to identify drug and syringe swap errors.

The aim of this study was to test the visual search efficacy of colour-coded compartmentalised trays to reduce anaesthetic drug administration errors compared with conventional trays under cognitive load. This was manipulated through a visuospatial working memory task in an online experiment. The experiment also tested whether the colour-coded trays influenced the relationship between participant confidence in responding and their response accuracy.^21^ We hypothesised that colour-coded trays elicit more accurate and faster error detection than conventional trays. Effects of colour-coded trays on confidence in responses were also tested.

## Methods

This study received ethical approval from the University of Derby (Reference ETH2021-4512; granted July 30, 2021) and Health Research Authority approval (Reference 21/HRA/1087; granted May 21, 2021). The experiment was accessed via weblink hosted by Psychopy Pavlovia (Psychopy, Nottingham, UK). Consent was obtained from all participants using an online form.

### Participants

We recruited clinical anaesthetists and operating department practitioners (ODPs) in any stage of training or practice through the National Institute for Health and Care Research (NIHR) Clinical Research Network (CRN) supported by the East Midlands NIHR CRN. Further participants were recruited using targeted online adverts (e.g. Twitter/X and LinkedIn). All participants had experience in the operating theatre and anaesthetic environment, and were aware of the specific drugs used in anaesthetic procedures and typical errors that might occur.

G-Power analyses^22^ revealed a minimum of 47 participants were required to detect a medium effect size (d=0.6) for drug detection errors (error-present *vs* error-absent) as a function of tray type (colour-coded *vs* conventional) with a power of 0.8, β=0.95 and α=0.05.

### Cognitive load error detection task

This was a 2×2 repeated-measures experimental design with tray type (colour-coded *vs* conventional trays) and drug error (error-present *vs* error-absent) as the independent variables. There were 16 error-present trials, eight for each tray condition (colour-coded, conventional). Errors included missing syringes, additional drugs, allergy risks, and syringes placed in an incorrect compartment/tray. All errors were matched across the tray conditions. There were also 20 error-absent trials, 10 for each tray condition. Trials were presented in a random order.

**Fig 1:**
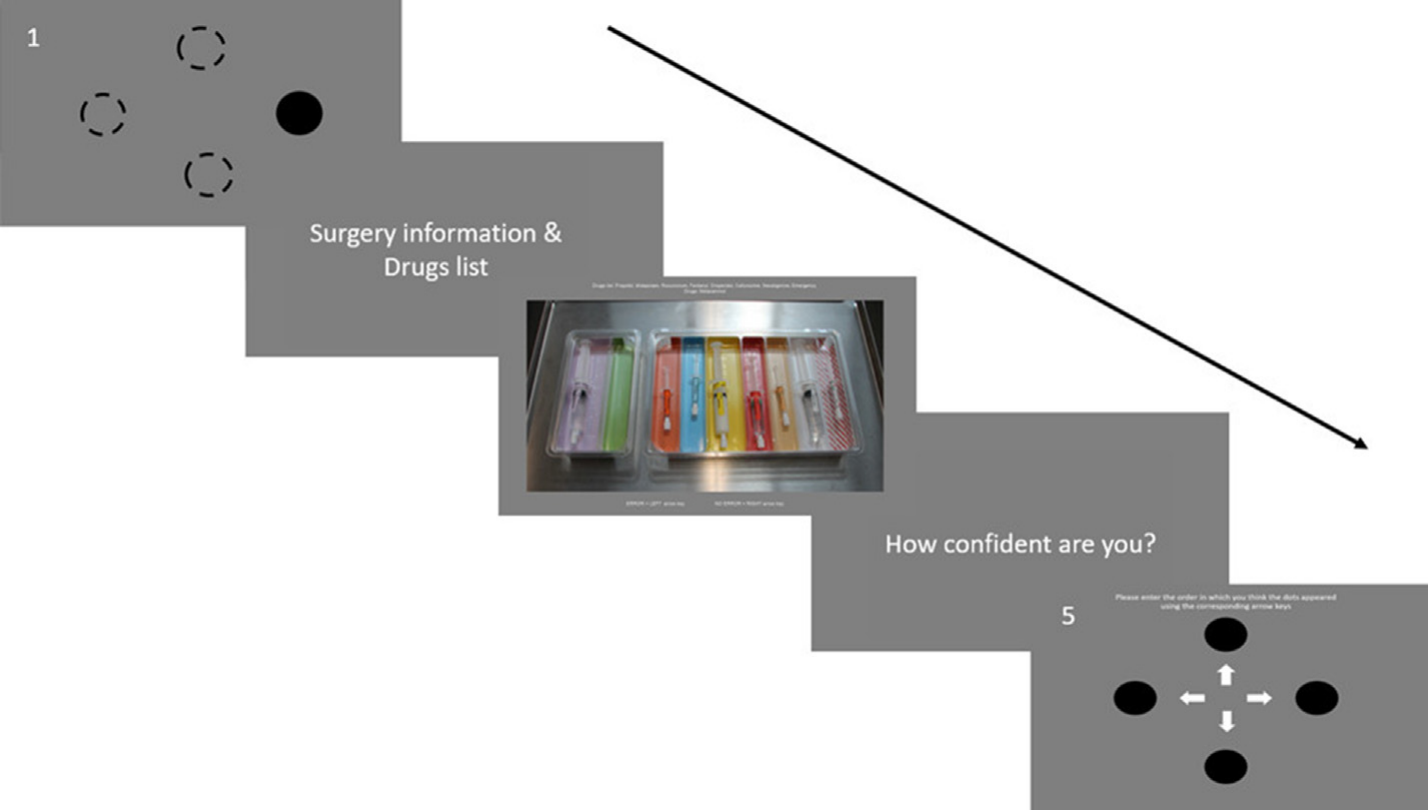
Schematic display of the experimental protocol demonstrating the wrap around cognitive load task [1,5]; vignette [2]; error detection task for the colour-coded tray [3]; and confidence rating [4]. The text in panel 3 provides a reminder of the relevant drugs list. The text in panel 5 states "Please enter the order in which you think the dots appeared using the corresponding arrow keys".

The trial process flow is displayed in Figure 1. Each trial included a different clinical vignette (designed by DH) describing routine clinical situations an anaesthetist would expect to encounter: for example, an Achilles tendon repair for a 37-yr-old female, in general good health, healthy weight range, and who has known penicillin allergy. Vignettes were accompanied by a trial-specific list of perioperative drugs: for example, propofol, atracurium, fentanyl, ondansetron, teicoplanin, neostigmine; and emergency drugs: atropine, ephedrine, metaraminol. An image of a loaded tray was then presented, and participants were required to conduct a visual search and indicate whether there was a drug error present with a mouse click (in the present example, co-amoxiclav was present instead of teicoplanin). There were 16 different vignettes for error-present trials. These were split into two groups of eight and counterbalanced between participants, such that half the participants completed error-present trials from Group 1 and the other half completed error-present trials from Group 2. There were 10 vignettes for error-absent trials, which were presented to all participants once for each tray type. Thus, all participants saw 18 different vignettes, which were counterbalanced across the two trays (36 trials in total).

To increase difficulty for the primary drug search task, a working memory-based cognitive load task was presented to all participants. This was used because in theatre settings visual search entails high cognitive demand and multitasking. The working memory task was presented in a wrap-around design, where participants were first asked to learn a sequence presented prior to the vignette and visual search task and then asked to recall the memory sequence after the search task and a confidence rating task (described below). The memory task increased the cognitive demand on the participants by requiring working memory capacity and therefore, to some extent, mimics the demands an anaesthetist might face when preparing and administering drugs.^16^^,^^17^

### Outcome measures

Accuracy, response times, and participant confidence scores for the error detection task were recorded, and accuracy scores were recorded for the cognitive load task. Confidence scores were used to calculate metacognitive sensitivity: the correlation between trial accuracy and trial confidence. Trials were marked correct if a drug error was correctly identified on error trials, and no drug error correctly identified on error-absent trials. The cognitive load task was given a recall accuracy score between 0 and 4 for each trial.

### Error detection under cognitive load task

The cognitive load task consisted of four black circles appearing sequentially in one of four cardinal locations randomised across all trials. Each circle was presented for 1 s. Once the sequence of four cardinal locations was complete, the screen moved automatically to display a surgical scenario and drugs list vignette. To allow the participant time to read at their own speed, this vignette screen was moved on by pressing the spacebar. The clinical vignette described a surgical procedure including key patient characteristics and a drugs list. Surgery type, patient characteristics, and error type were counterbalanced.

**Fig 2:**
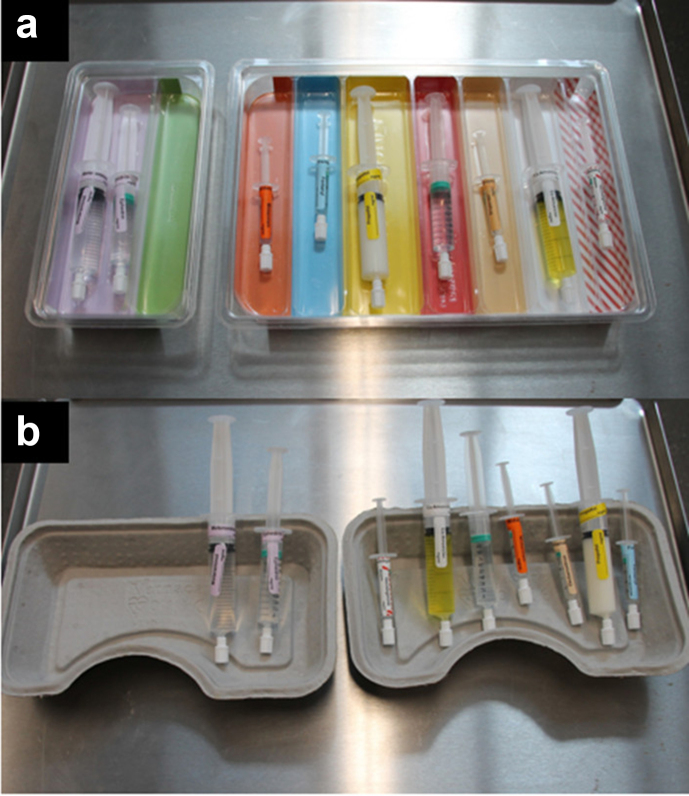
Snapshot of (a) the colour-coded tray and (b) the conventional tray.

The next phase was the presentation of the drug tray image where participants were required to detect if an error was present, again triggered with a press of the spacebar to move from the vignette to the error detection task. Here, an image of either a conventional single-compartmented, grey, multipurpose paper mulch tray or loaded colour-coded tray was presented with the drugs list (Fig. 2). The colour-coded tray was organised by drug class matching the ISO26825:2020 international colour-coded labelling system (Rainbow Tray™; Uvamed, Loughborough, UK). For all trials, the relevant drugs list was presented at the top of the screen. Participants pressed the keyboard left arrow key if an error was present or the keyboard right arrow key if there was no error.

The experiment progressed when a participant error-present or error-absent response was given. Participants then provided a confidence score, from 1 (not very confident) to 5 (very confident), for their error detection response. The cognitive load recall task was then presented, this was four circles and arrows corresponding to the direction of the circle presented. Here the participant used the arrow keys to recall the exact sequence order the circles had appeared in (left, right, down, up). Once four responses had been recorded, the trial ended.

Between each trial, a rest screen was presented to participants. This was manually moved on with the spacebar. Thus, there were 36 different trials for each participant.

Drug scenario images were taken on a Canon EOS camera, with 35 mm focal length, positioned on a tripod above the drug tray on a medical trolly.

The study was created using Psychopy (Nottingham, UK) and hosted on their online platform Pavlovia (Open Science Tools Limited, https://pavlovia.org/docs/home/about).

### Procedure

Participants were sent a hyperlink to the online experiment. This opened a Qualtrics survey, where participants could read the study information sheet, sign an electronic consent form, and complete demographic questions. Participants were then presented with the experiment instructions and a link to the experiment on the online platform Pavlovia.

### Statistical analysis plan

Trials with correct responses were subjected to a tray (colour-coded *vs* conventional) × drug-error presence (error-present *vs* error-absent) repeated-measures analysis of variance (anova). A log transformation corrected skewness in response times. Log transformed times for correct responses were analysed with a 2×2 repeated-measures anova.

Metacognitive sensitivity was calculated from accuracy of responses and confidence scores. This involved subtracting the mean confidence score from the mean accuracy rate. Negative scores indicate under-confidence, and positive scores indicate overconfidence.^23^ Metacognitive sensitivity scores were entered into a tray (colour-coded *vs* conventional) and drug-error (error-present *vs* error-absent) repeated-measures anova. The relationship between confidence ratings and response accuracy for each condition were tested with Pearson's correlation.

The design also allowed for signal detection measures. These included *d′* (a measure of sensitivity to the error/signal) and *c* (the criterion bias to say ‘yes’ regardless of the drug error condition), as measures of drug error detection performance. A hit was recorded when participants correctly identified an error. A false alarm was recorded when participants identified an error on an error-absent trial. Signal detection analysis was conducted using the z-scores of hits (zHits) and the z-scores of False Alarms (zFalse Alarms). By comparing the values of zHits – zFalse Alarms for each tray type, sensitivity to the error signal was tested. The response bias towards indicating that there was an error irrespective of the stimuli was tested using the values derived from (zHits + zFalse Alarms)/2 for each tray type.

## Results

### Participants

There were 53 complete responses. Of these (mean [range] age: 40 [23–61] yr, 22 female), 32 were consultant anaesthetists (11 female, mean experience 16.7 [7.4] yr), six were trainee anaesthetists (three female, mean experience 2.8 [1.5] yr), four were ODPs (four female, mean experience 18.9 [7.9] yr), and 11 were trainee ODPs (six female, mean experience 1.1 [1.6] yr). One participant's data were more than three standard deviations below the mean; as an outlier their data were removed.

### Drug error detection task

#### Accuracy

**Fig 3:**
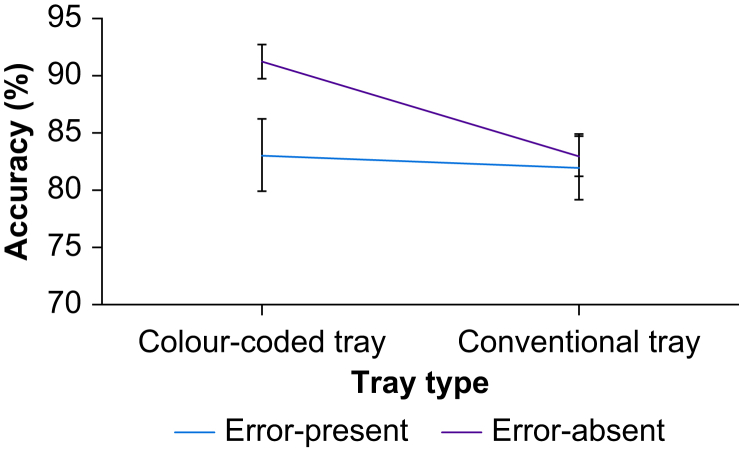
Mean percentage of correct responses.

Trials were explored in a tray (colour-coded *vs* conventional) × drug-error (error-present *vs* error-absent) repeated-measures anova. The main effect of tray was significant (*F*[51]=8.17, MSe=0.013, *P*=0.006, $\eta_{p}^{2}$ =0.14). Participants detected errors more often in colour-coded trays than in conventional trays. There was no significant effect of drug error (error *vs* no error) (*F*[51]=2.91, MSe=0.050, *P*=0.094, $\eta_{p}^{2}$ =0.05). There was a significant interaction between tray type and drug-error (*F*[51]=4.38, MSe=0.010, *P*=0.041, $\eta_{p}^{2}$ =0.08) (Fig. 3). *Post hoc* Bonferroni corrected *t*-tests (*p*<0.0125) revealed that error-absent trials had more accurate response rates in colour-coded trays compared with conventional trays (*t*[51]=4.12, *P*<0.001, Cohen's d=0.57). Responses in error-absent trials were not reliably more accurate than error-present trials in colour-coded trays (*t*[51]=–2.44, *P*=0.018, Cohen's d=0.36) (Table 1). There was also no difference between colour-coded trays and conventional trays for error-present trials (*P*=0.65), or between error-present and error-absent trials for conventional trays (*P*=0.78).

**Table 1:** Mean percentage correct response and response times as a function of tray type.

Error type	Tray type			
	Colour-coded tray correct response,% (sd)	Conventional tray correct response,% (sd)	Colour-coded tray response time, s (sd)	Conventional tray response time, s (sd)
Error-present	83.0 (22.4)	81.3 (20.5)	12.1 (5.0)	12.0 (5.7)
Error-absent	91.1 (10.5)	83.8 (11.7)	17.4 (7.8)	18.7 (9.2)
Total mean	87.0 (18.0)	82.5 (16.7)	14.7 (7.0)	15.3 (8.3)

#### Response times

**Fig 4:**
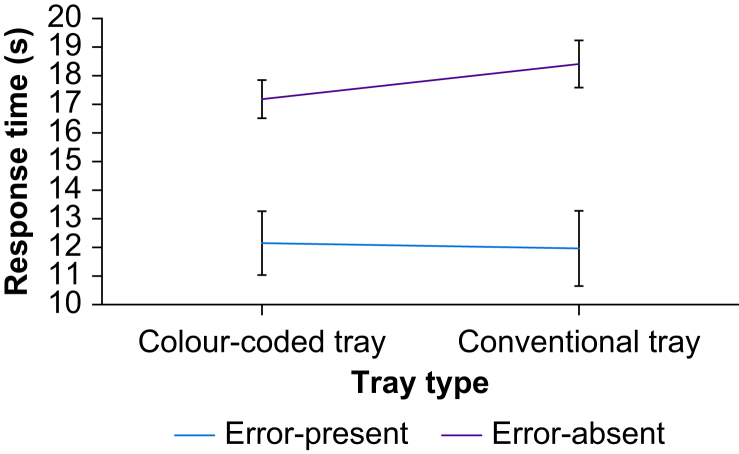
Mean response time in seconds for correctly identified trials.

Trials were explored in a tray (colour-coded *vs* conventional) × drug-error (error-present *vs* error-absent) repeated-measures anova (Fig. 4). The main effect of tray on response times was not significant (*F*[51]=0 .015, MSe=0.010, *P*=903, $\eta_{p}^{2}$ <0.01). Correct responses to error-present trials were faster than correct responses to error-absent trials (*F*[51]=78.56, MSe=0.028, *P*<0.001, $\eta_{p}^{2}$ =0.61). There was also a tray by error interaction (*F*[51]=6.94, MSe=0.006, *P*=0.011, $\eta_{p}^{2}$ =0.12), driven by faster responses to error-absent trials in the colour-coded tray condition compared with the conventional tray (Table 1), although comparisons did not pass the Bonferroni threshold (*p*<0.0125) for significance (*t*[51]=–2.131, *p*=0.038, Cohen's d=0.32). There were, however, significant differences between error-present and error-absent trials in colour-coded trays (*t*[51]=–6.21, *P*<0.001, Cohen's d=–0.85) and between the two error categories in the conventional tray (*t*[51]=–7.50, *P*<0.001, Cohen's d=–0.77). The difference for error-present trials between colour-coded trays and conventional trays was not significant (*P*=0.67).

#### Confidence ratings

The main effect of tray on confidence rating was not significant (*F*[51]=2.83, MSe=127.57, *P*=0.098, $\eta_{p}^{2}$ =0.05). However, the main effect of drug-error presence (*F*[51]=10.77, MSe=504.50, *P*=0.002, $\eta_{p}^{2}$ =0.17) demonstrated that participants were under-confident when responding to error-present trials across both tray types (–9.76 [sd 17.34]) compared with error-absent trials (0.458 [sd 16.73]). The interaction between tray and error presence was not significant (*F*[51]=1.91, MSe=114.45, *P*=0.173, $\eta_{p}^{2}$ =0.04).

There was a strong positive relationship between accuracy of responses and confidence scores for colour-coded trays (*r*[52]=0.696, *P*<0.001) and a moderate positive relationship for conventional trays (*r*[52]=0.447, *P*<0.001). This indicated a stronger correlation between confidence and task performance for colour-coded compartmentalised trays, indicative of good metacognitive sensitivity and accurate perception of performance.

#### Signal detection analysis

Signal detection analysis tests participants' ability to detect errors and avoid response bias (to check whether participants responded that there was an error present irrespective of trial content). There was a significant difference between *d’* scores (a measure of sensitivity to the signal; zHits – zFalse Alarms) of the two trays, with better sensitivity to the error signal for colour-coded trays (2.28 [sd 0.90]) compared with conventional trays (1.50 [sd 1.15]) (*t*[51]=4.80, *P*<0.001, Cohen's d=0.67). There was also a significant difference between *c* ratings (the criterion bias to say ‘yes’ regardless of the information); (zHits + zFalse Alarms)/2), with colour-coded trays (–1.68 [sd 0.61]) scoring lower than conventional trays (–1.25 [sd 0.55]) (*t*[51]=–4.38, *P*<0.001, Cohen's d=0.61), indicating reduced response bias for colour-coded trays.

## Discussion

We compared visual search efficacy for colour-coded trays and conventional trays under cognitive load. Colour-coded trays were superior for the accuracy of visual search of loaded drug trays, and elicited more accurate responses to drug errors than conventional trays. Signal detection measures indicated more sensitivity to signals in colour-coded trays and a reduced criterion to respond that there was an error irrespective of presented information.

The superiority of responses using colour-coded trays was consistent with other visual search research where colour coding and standardisation improve outcomes.^24^^,^^25^ Our findings support recent national recommendations made on the handling of injectable medicines that colour-coded trays are a short-term technological solution to improve medicines safety.^26^ The results support existing research into colour-coded templates and trays organised by colour and location, indicating that colour coding can facilitate performance.^27^, ^28^, ^29^, ^30^ The trays used in previous studies have shown advantages for mitigating syringe-type drug errors. With improved sensitivity to errors in colour-coded compartmentalised trays, introducing these trays into anaesthetising locations could mitigate some drug-related errors in anaesthetic drug administration (e.g. syringe swaps or misidentifications).

The safety benefits arising from colour coding of drug trays is consistent with the literature on colour-coded labelling of individual drug syringes. Correct recognition of colour-labelled drug syringes is faster than when monochrome labels or drug ampules are used.^31^ Similarly, increasing colour-related cues during drug administration as a component of a systems approach to improving medication handling has been shown in pragmatic clinical trials to reduce drug-swap errors.^32^

Our study also demonstrates that colour-coded trays helped participants show greater metacognitive sensitivity, such that they were more confident about their correct responses than their errors. Colour acts as a guide during visual search, with early visual features (e.g. colour) guiding visual attention,^33^^,^^34^ but relies on standardised colour coding by manufacturers. These findings further support standardised colour coding for vial tops to mitigate medication error.^35^^,^^36^

The search benefit identified in this study is primarily driven by responses to error-absent trials. Error-absent trials for conventional trays were identified more slowly and less often, with more ‘false-positive’ responses (reporting an error when there was none). In visual search, trials without a target can be problematic. The decision to terminate a search when a target has been found is clear, whereas the decision to terminate a search when a target is absent is more ambiguous.^37^^,^^38^ Ending a search early can result in targets being missed, whereas prolonging search for non-existent errors can create issues with speed and efficiency.^39^^,^^40^

### Challenges and future directions

To ensure experimental controls and a fair comparison, syringes in the conventional trays were neatly laid out side-by-side within the tray, with all labels visible, a more favourable scenario than would typically occur in the real world. It is possible that positive results for colour-coded trays might have been underestimated because of the artificial neatness of the syringe presentation in conventional trays. It should be noted that this was a screen-based task rather than a clinical task. The findings compliment those of an eye-tracking experiment testing colour-coded trays,^19^ and corroborate subjective reports from anaesthetists about the same tray design.^30^ Future research should test a more realistic layout of syringes in conventional trays compared with colour-coded trays to gain an understanding of the effect of tray organisation. This could be achieved in an eye-tracking study during simulated anaesthetic environments with anaesthetists using different tray types.

### Conclusions

Our results in a simulated environment favoured colour-coded trays, which offered search advantages over conventional trays in error detection and identification of correctly loaded trays. This supports previous findings that colour-coded trays improve visual search efficiency, providing further evidence that introducing colour-coded trays can provide safety mitigations in anaesthesia.

## Authors’ contributions

Study conception and design: all authors

Development of materials for the study: EJNS, VL, FAM, DWH

Data collection and analysis: VL

Writing of the manuscript: all authors

All authors revised the manuscript for significant intellectual content, gave approval for the version to be published, and agreed to be accountable for the work in ensuring that questions related to the accuracy or integrity of any part of the work are appropriately investigated and resolved.

## Acknowledgements

With thanks to Ann Bates, Amy Cauldwell, University of Derby ODP students, Sophie Rutherford, John Shacklock, and the East Midlands Clinical Research Network.

## Declarations of interest

DWH accepts fees for advising in civil, criminal, and coronial medicolegal cases and is a member of the associate editorial board of the *British Journal of Anaesthesia*. The remaining authors declare that they have no competing interests. None of the authors have any financial interest in sales of the company selling the trays (Uvamed, Leicester, UK), which provided free samples of the trays during the design of the study. Rainbow Trays™ are available commercially and were provided by the company using grant funding. The funder and Uvamed had no involvement in the design, conduct, analysis, or interpretation of this study.

## Funding

Innovate UK by a grant (no. 44612) shared between University of Derby and Uvamed, UK.
